# Supplementary material for: Physiological and Growth Responses of Thai Rice Genotypes to High Salinity Stress at the Seedling and Reproductive Stages
Source: Plants (Basel). 2025 Dec 9;14(24):3748. doi: 10.3390/plants14243748 (PMC12737130; doi:10.3390/plants14243748)
Supplement: Supplementary file 1 [file plants-14-03748-s001.zip › plants-3991667-supplementary.pdf]

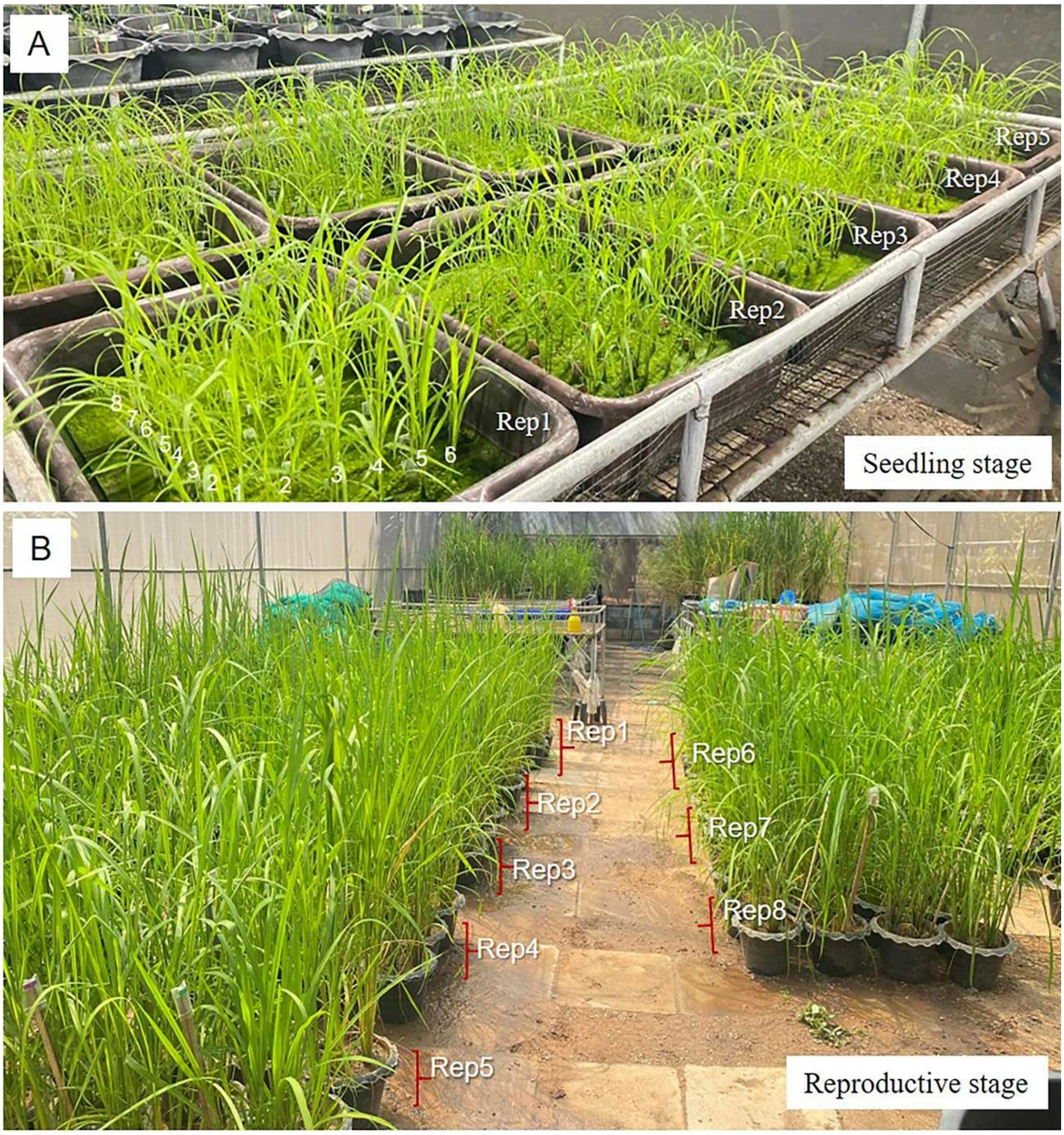

Figure S1. Rice plants at seedling (**A**) and reproductive stage (**B**) before applying salinity treatment. All rice plants grew under natural light in the green house at Department of Biology, Faculty of Science, Khon Kaen University.

Table S1. The salt injury scores, SPAD, plant height and total biomass of 24 rice genotypes grown under the non-saline and saline condition. The significant difference ($p < 0.05$ and $p < 0.01$) among the rice genotypes in both salinity conditions are denoted with different lowercase letters. Mean % changes from control values are displayed, with significant differences between control and salt stress treatments are denoted by *. Data are Mean ± SE (n = 5).

Rice genotype	Score value of stress plants		SPAD			Plant height (cm)			Total biomass (g DW plnat^{-1})		
	7 DAS	10 DAS	Control	Stress	%	Control	Stress	%	Control	Stress	%
Go Main Surin (GMS)	4.6 b–f	4.6 f–j	31.72 ab	28.04 a–d	−11	64.20 cd	37.90 k–n	−40*	0.34 f–j	0.15 o–r	−55*
Gon Gaew (GG)	4.6 b–f	6.6 b–d	32.08 ab	18.22 ef	−44*	61.60 de	35.90 l–o	−42*	0.39 c–i	0.13 qr	−66*
HMLN	5.2 b–d	7.4 b	33.18 a	19.60 ef	−41*	44.60 h–k	27.96 q	−37*	0.22 m–o	0.10 r	−55*
Hom Dang Nouy (HDN)	3.0 h	3.2 j	31.82 ab	29.82 a–c	−6	65.70 cd	36.10 l–o	−44*	0.36 e–j	0.13 qr	−59*
Hom Noun (HN)	3.0 h	4.4 f–j	30.72 ab	29.66 a–c	−3	71.26 ab	42.90 i–k	−40*	0.47 ab	0.16 n–r	−64*
IR29	7.0 a	9.0 a	30.26 a–c	7.52 g	−76*	44.70 h–k	27.10 q	−38*	0.18 n–q	0.10 r	−46*
LLR395	3.2 gh	4.4 f–j	34.34 a	30.12 a–c	−12	48.60 g–i	31.28 o–q	−35*	0.33 h–k	0.15 o–r	−52*
KDML105	4.6 b–f	6.6 b–d	32.10 ab	18.18 ef	−42*	64.20 cd	35.70 l–o	−44*	0.40 b–g	0.12 qr	−67*
Khao Gaew (KG)	3.6 e–h	3.6 h–j	31.30 ab	28.62 a–d	−9	69.00 a–c	39.36 k–m	−43*	0.40 b–h	0.15 o–r	−62*
Khao Supan (KS)	4.8 b–e	5.0 e–h	31.14 ab	24.60 b–e	−21	74.40 a	46.80 g–j	−37*	0.44 a–d	0.14 o–r	−67*
Lou Tang (LT)	5.8 ab	7.4 b	31.14 ab	14.68 fg	−53*	62.20 de	38.04 k–n	−38*	0.30 j–l	0.12 qr	−54*
Leuang Puang Tawng (LPT)	3.6 e–h	3.6 h–j	31.16 ab	30.90 ab	−1	68.30 bc	43.50 i–k	−36*	0.42 a–f	0.16 n–r	−57*
Luang Pratahn (LP)	5.2 b–d	7.4 b	31.06 ab	15.56 f	−52*	65.10 cd	36.90 l–n	−42*	0.38 d–j	0.14 p–r	−60*
Leuang Tah Yang (LTY)	5.4 bc	7.0 bc	29.78 a–c	15.98 f	−47*	66.90 b–d	37.80 k–n	−43*	0.38 d–i	0.12 qr	−68*
Ma Hom (MH)	5.4 bc	6.6 b–d	29.58 a–d	21.30 d–f	−27	57.70 ef	33.74 n–p	−41*	0.33 g–k	0.12 qr	−63*
Mali	5.2 b–d	6.2 b–e	30.84 ab	22.10 c–f	−29	64.60 cd	34.90 m–p	−45*	0.33 g–k	0.11 qr	−68*
Puang Tawng (PT)	4.4 c–g	4.2 g–j	31.96 ab	29.16 a–d	−8	68.50 bc	39.30 k–m	−42*	0.39 d–i	0.16 n–r	−54*
Pahn Tawng60 (PT60)	3.4 f–h	4.8 e–i	31.64 ab	27.94 a–d	−11	69.20 a–c	39.20 k–m	−43*	0.48 a	0.16 o–r	−65*
Pahn Tawng59 (PT59)	4.0 d–h	5.4 d–g	31.70 ab	28.70 a–d	−9	69.08 a–c	40.60 j–l	−41*	0.42 a–e	0.15 o–r	−63*
Pokkali (PK)	3.4 f–h	3.4 i–j	30.88 ab	32.84 a	7	71.60 ab	50.40 f–h	−28*	0.47 a–c	0.21 m–p	−47*
PTT1	4.6 b–f	6.6 b–d	30.92 ab	19.02 ef	−38*	53.10 fg	29.86 pq	−43*	0.26 k–m	0.12 qr	−54*
RD61	4.2 c–h	5.8 c–f	32.76 ab	19.98 ef	−41*	47.90 g–j	30.10 pq	−37*	0.32 i–k	0.14 p–r	−56*
RD73	3.2 gh	4.2 g–f	30.82 ab	28.40 a–d	−8	64.30 cd	34.50 m–p	−46*	0.38 d–i	0.14 p–r	−64*
TSKC1−144	5.8 ab	7.4 b	32.60 ab	16.76 ef	−49*	65.00 cd	36.10 l–o	−40*	0.23 l–n	0.12 qr	−49*
Mean	4.5**	5.6**	31.48**	23.24	−26	62.57**	36.91	−40	0.36**	0.14	−59

Table S2. Photosynthetic pigments in flag leaves including SPAD index, total chlorophyll, chlorophyll a, chlorophyll b, and chlorophyll a/b of ten rice genotypes. Plants were grown under control condition and salt stress (irrigation with 150 mM NaCl instead of water for 19 days during the reproductive stage). Different lowercase letters indicate significant differences ($p < 0.05$ and $p < 0.01$) among rice genotypes and between salinity treatments. Mean % changes from control values are displayed, with significant differences between control and salt stress treatments are denoted by *. Data are Mean ± SE (n = 4).

Rice genotype	SPAD			Total chlorophyll (mg g^{-1} FW)			Chlorophyll a (mg g^{-1} FW)			Chlorophyll b (mg g^{-1} FW)			Chlorophyll a/b		
	Control	Stress	%	Control	Stress	%	Control	Stress	%	Control	Stress	%	Control	Stress	%
GMS	37.72 b–c	36.30 d–g	−4*	3.221 a	2.763 a	−14	1.422 a	1.387 a	−2	1.799 a	1.376 a	−22	0.809 a	1.239 a	56
HDN	37.32 c–f	38.67 b–d	4	3.299 a	3.050 a	−7	1.436 a	1.456 a	1	1.863 a	1.594 a	−12	0.804 a	0.923 a	17
HN	36.30 d–g	35.62 e–g	−2	3.064 a	2.768 a	−9	1.475 a	1.387 a	−6	1.589 a	1.380 a	−12	0.951 a	1.097 a	15
LLR395	42.25 a	39.82 ab	−6	3.360 a	3.177 a	−5	1.391 a	1.431 a	3	1.968 a	1.745 a	−10	0.730 a	0.846 a	17
KDM	36.17 e–g	35.32 fg	−2	3.154 a	2.990 a	−5	1.429 a	1.428 a	0	1.724 a	1.561 a	−7	0.850 a	0.920 a	12
KG	35.45 fg	37.92 b–e	7*	2.953 a	3.394 a	16	1.469 a	1.354 a	−8	1.483 a	2.039 a	48	1.078 a	0.674 a	−32
LPT	36.52 c–g	37.37 c–f	2	3.219 a	3.076 a	−2	1.467 a	1.448 a	−1	1.752 a	1.628 a	3	0.954 a	0.894 a	4
PK	37.97 b–e	38.85 bc	2	3.086 a	3.029 a	0	1.418 a	1.434 a	1	1.668 a	1.594 a	4	0.909 a	0.915 a	12
RD73	35.62 e–g	37.55 b–f	5	3.059 a	3.259 a	10	1.480 a	1.422 a	−4	1.578 a	1.836 a	30	1.032 a	0.785 a	−14
TSKC1–144	36.32 d–g	34.22 g	−6	3.129 a	2.678 a	−14	1.445 a	1.435 a	−1	1.683 a	1.243 a	−24	0.882 a	1.166 a	36
Mean	37.16	37.16	0	3.154	3.018	−3	1.443	1.418	−2	1.711	1.599	0	0.900	0.946	12

Table S3. The effective quantum yield of PSII photochemistry (Φ PSII) and electron transport rate (ETR) of ten rice genotypes when illuminated with light intensity at 1,200 μmol (photon) m^{-2} s^{-1}. Plants were grown under control condition and salt stress (irrigation with 150 mM NaCl instead of water for 14 days during the reproductive stage). Different lowercase letters indicate significant differences ($p < 0.05$ and $p < 0.01$) among rice genotypes and between salinity treatments. Mean % changes from control values are displayed, with significant differences between control and salt stress treatments are denoted by *. Data are Mean ± SE (n = 4).

**Rice genotype**	**Φ PSII**			**ETR (μmol e^{-1} m^{-2} s^{-1})**		
	**Control**	**Stress**	**%**	**Control**	**Stress**	**%**
GMS	0.147 g–j	0.170 e–i	16	77.45 g–i	89.60 e–h	16
HDN	0.216 a–c	0.153 f–j	−27*	113.64 bc	80.56 e–i	−27*
HN	0.189 c–f	0.185 c–g	2	99.26 c–e	97.54 c–f	2
LLR395	0.211 b–d	0.172 e–i	−16*	111.10 b–d	90.62 e–h	−16*
KDM	0.178 c–h	0.168 e–j	−4	93.46 d–g	88.59 e–h	−4
KG	0.229 ab	0.129 j	−43*	120.59 ab	68.12 i	−43*
LPT	0.253 a	0.146 h–j	−41*	133.23 a	76.61 g–i	−41*
PK	0.162 e–j	0.136 ij	−16	85.11 e–i	71.79 hi	−16
RD73	0.172 d–i	0.155 f–j	−9	90.87 e–h	81.85 e–i	−10
TSKC1–144	0.186 c–g	0.200 b–e	10	98.06 c–f	78.85 f–i	−18
Mean	0.194**	0.162	−13	102.28**	82.42	−16

Table S4. Leaf gas exchange including net photosynthesis rate (Pn), stomatal conductance (gs) and transpiration rate (Tr) were investigated under PAR of 1,200 μmol (photon) m^{-2} s^{-1} in flag leaves of ten rice genotypes. Plants were grown under control condition and salt stress (irrigation with 150 mM NaCl instead of water for 14 days during the reproductive stage). Different lowercase letters indicate significant differences ($p < 0.05$ and $p < 0.01$) among rice genotypes and between salinity treatments. Mean % changes from control values are displayed, with significant differences between control and salt stress treatments are denoted by *. Data are Mean ± SE (n = 4).

Rice genotype	Pn (μmol CO_2 m^{-2} s^{-1})			gs (mol H_2O m^{-2} s^{-1})			Tr (mmol H_2O m^{-2} s^{-1})		
	**Control**	**Stress**	**%**	**Control**	**Stress**	**%**	**Control**	**Stress**	**%**
GMS	14.01 bc	5.093 ef	−64*	0.203 a−c	0.059 f−h	−70*	5.478 ab	1.905 de	−65*
HDN	10.04 d	3.20 f	−68*	0.133 c−f	0.042 gh	−68*	4.729 a−c	1.507 de	−68*
HN	11.54 b−d	4.77 f	−71*	0.192 a−c	0.051 gh	−58*	4.448 bc	1.709 de	−55*
LLR395	17.54 a	3.23 f	−81*	0.212 ab	0.038 gh	−82*	5.900 a	1.521 de	−74*
KDML105	12.10 b−d	6.07 ef	−48*	0.246 a	0.062 e−h	−74*	4.703 a−c	1.975 d	−53*
KG	11.07 cd	5.37 ef	−49*	0.098 d−h	0.041 gh	−55	4.145 c	1.362 de	−68*
LPT	13.30 bc	4.25 ef	−67*	0.109 d−g	0.026 h	−75*	4.200 c	0.719 e	−82*
PK	12.05 b−d	3.50 f	−67*	0.136 b−e	0.054 gh	−59*	4.166 c	1.847 de	−55*
RD73	14.36 ab	6.80 e	−49*	0.212 ab	0.060 e−h	−68*	5.219 a−c	1.991 d	−60*
TSKC1−144	13.05 b−d	5.44 ef	−58*	0.143 b−d	0.049 gh	−65*	4.517 bc	1.757 de	−61*
Mean	12.91**	4.77	−62	0.169**	0.048	−67	4.751**	1.629	−64

Table S4 (cont.). Intrinsic water use efficiency (WUEi), water use efficiency (WUE), and intercellular CO_2 per ambient CO_2 (Ci/Ca) in flag leaves of ten rice genotypes. Plants were grown under control condition and salt stress (irrigation with 150 mM NaCl instead of water for 14 days during the reproductive stage). Different lowercase letters indicate significant differences ($p < 0.05$ and $p < 0.01$) among rice genotypes and between salinity treatments. Mean % changes from control values are displayed, with significant differences between control and salt stress treatments are denoted by *. Data are Mean ± SE (n = 4).

Rice genotype	WUEi (μmol CO_2 mol H_2O^{-1})			WUE (μmol CO_2 mmol H_2O^{-1})			Ci/Ca		
	**Control**	**Stress**	**%**	**Control**	**Stress**	**%**	**Control**	**Stress**	**%**
GMS	69.28 ef	83.30 c–f	17	2.57 b–e	2.39 c–e	−9	0.675 a	0.632 a–d	−7
HDN	77.59 d–f	70.72 ef	−2	2.08 de	1.92 e	−8	0.633 a–c	0.674 ab	10
HN	90.17 b–f	64.61 f	6	2.90 b–e	1.935 e	−28	0.590 a–f	0.693 a	25
LLR395	83.09 c–f	93.87 b–f	11	3.03 b–e	2.780 b–e	−13	0.617 a–e	0.551 a–f	−11
KDML105	51.96 f	131.48 bc	149*	2.58 b–e	3.519 bc	40	0.748 a	0.386 fg	-49*
KG	117.09 b–e	138.64 ab	23	2.79 b–e	3.66 b	44	0.465 b–f	0.397 fg	−9
LPT	126.61 bc	181.05 a	41*	3.28 bc	6.526 a	97*	0.429 d–g	0.240 g	−50
PK	86.11 c–f	66.76 f	−18	2.79 b–e	1.959 e	−26	0.610 a–e	0.682 a	14
RD73	69.11 ef	120.35 b–d	73	2.71 b–e	3.570 b	32	0.675 a	0.464 c–f	−32*
TSKC1–144	92.54 b–f	125.65 b–d	36	2.94 b–e	3.240 b–d	16	0.576 a–f	0.418 e–g	−27
Mean	86.35	107.65	17	2.77	3.15	15	0.602	0.514	−14

Table S5. Relative water content (RWC), osmotic potential (OP) and osmotic adjustment (OA) in flag leaves of ten rice genotypes. Plants were grown under control condition and salt stress (irrigation with 150 mM NaCl instead of water for 19 days during the reproductive stage). Different lowercase letters indicate significant differences ($p < 0.05$ and $p < 0.01$) among rice genotypes and between salinity treatments. Mean % changes from control values are displayed, with significant differences between control and salt stress treatments are denoted by *. Data are Mean ± SE (n = 4).

**Rice genotype**	**RWC (%)**			**OP (MPa)**			**OA (MPa)**
	**Control**	**Stress**	**%**	**Control**	**Stress**	**%**	**Stress**
GMS	90.84 a–d	89.27 a–e	−2	−1.346 a	−2.757 d–f	107*	2.380 ab
HDN	92.28 a–c	90.07 a–d	−2	−1.278 a	−2.025 b	59*	1.778 d
HN	91.15 a–c	87.23 c–e	−4	−1.290 a	−2.219 bc	72*	1.873 cd
LLR395	93.97 a	93.52 a	0	−1.275 a	−2.605 c–e	106*	2.401 ab
KDML105	91.20 a–c	92.17 a–c	1	−1.357 a	−2.517 c–e	85*	2.283 a–c
KG	91.41 a–c	85.20 de	−7*	−1.298 a	−3.149 f	143*	2.576 a
LPT	94.16 a	88.12 b–e	−6*	−1.334 a	−2.945 ef	122*	2.541 a
PK	91.49 a–c	87.52 b–e	−4	−1.299 a	−1.886 b	46*	1.598 de
RD73	92.62 a–c	84.70 e	−9*	−1.288 a	−2.162 bc	68*	1.760 d
TSKC1–144	93.03 ab	88.90 a–e	−4	−1.416 a	−2.309 b–d	64*	2.002 b–d
Mean	92.21	88.67	−4	−1.32	−2.46**	87	2.12**

Table S6. Electrolyte leakage (EL) and malondialdehyde (MDA) in flag leaves of ten rice genotypes. Plants were grown under control condition and salt stress (irrigation with 150 mM NaCl instead of water for 19 days during the reproductive stage). Different lowercase letters indicate significant differences ($p < 0.05$ and $p < 0.01$) among rice genotypes and between salinity treatments Mean % changes from control values are displayed, with significant differences between control and salt stress treatments are denoted by *. Data are Mean ± SE (n = 4).

**Rice genotype**	**EL (%)**			**MDA (nmol g^{-1} FW)**		
	**Control**	**Stress**	**%**	**Control**	**Stress**	**%**
GMS	7.81 cd	12.61 a–c	71	25.37 d	24.13 d	−3
HDN	8.61 cd	9.64 b–d	22	29.15 cd	39.11 a–c	2
HN	8.55 cd	15.11 a	78*	34.81 b–d	49.21 a	40*
LLR395	9.08 b–d	11.62 a–d	38	27.87 cd	28.84 cd	5
KDML105	7.19 d	7.82 cd	11	34.82 b–d	35.31 b–d	3
KG	11.61 a–d	15.13 a	31	27.06 cd	25.34 d	5
LPT	9.43 b–d	14.44 ab	53	24.13 d	24.47 d	2
PK	7.46 cd	9.36 b–d	29	26.66 cd	28.23 cd	11
RD73	10.61 a–d	8.22 cd	−17	28.05 cd	34.58 b–d	28
TSKC1–144	8.75 cd	9.14 b–d	18	36.06 a–d	46.60 ab	27
Mean	8.91	11.31*	33	29.40	33.58	12

Table S7. Contents of sodium ion (Na^+), potassium ion (K^+) and ratio of sodium to potassium ion (Na^+/K^+) in flag leaves of ten rice genotypes. Plants were grown under control condition and salt stress (irrigation with 150 mM NaCl instead of water for 19 days during the reproductive stage). Different lowercase letters indicate significant differences ($p < 0.05$ and $p < 0.01$) among rice genotypes and between salinity treatments. Mean % changes from control values are displayed, with significant differences between control and salt stress treatments are denoted by *. Data are Mean ± SE (n = 4).

Rice genotype	Na^+ content (%)			K^+ content (%)			Na^+/K^+ ratio		
	Control	Stress	Values increased from control (time)	Control	Stress	%	Control	Stress	Values increased from control (time)
GMS	0.060 h	2.39 cd	40*	1.560 c–f	1.250 fg	–16	0.039 h	1.921 a	50*
HDN	0.050 h	1.68 f	36*	1.893 b–d	1.358 e–g	–29*	0.027 h	1.251 e	50*
HN	0.033 h	2.67 ab	84*	1.848 b–d	1.873 b–d	0	0.018 h	1.510 cd	86*
LLR395	0.043 h	2.72 a	65*	1.763 cd	2.688 a	55*	0.027 h	1.025 f	43*
KDML105	0.050 h	2.46 a–c	61*	1.340 e–g	1.338 e–g	0	0.038 h	1.836 ab	63*
KG	0.040 h	2.43 b–d	69*	1.313 fg	1.540 d–f	18	0.030 h	1.649 bc	64*
LPT	0.038 h	2.72 a	77*	1.113 g	1.530 d–f	38*	0.033 h	1.783 ab	56*
PK	0.058 h	1.09 g	19*	1.730 c–e	2.218 b	30*	0.034 h	0.491 g	15*
RD73	0.060 h	2.16 de	36*	1.850 b–d	1.553 c–f	–16	0.032 h	1.407 de	43*
TSKC1–144	0.048 h	1.98 e	44*	1.923 b–d	1.953 bc	7	0.025 h	1.023 f	43*
Mean	0.048	2.230**	53	1.633	1.730**	9	0.030	1.390**	51

Table S8. Total sugar and starch content in the flag leaves of ten rice genotypes. Plants were grown under control condition and salt stress (irrigation with 150 mM NaCl instead of water for 19 days during the reproductive stage). Different lowercase letters indicate significant differences ($p < 0.05$ and $p < 0.01$) among rice genotypes and between salinity treatments. Mean % changes from control values are displayed, with significant differences between control and salt stress treatments are denoted by *. Data are Mean ± SE (n = 4).

**Rice genotype**	**Total sugar (mg g^{-1} FW)**			**Starch (mg g^{-1} FW)**		
	**Control**	**Stress**	**%**	**Control**	**Stress**	**%**
GMS	23.99 a–c	18.80 b–f	107	6.94 a–d	8.66 a–c	28
HDN	16.14 c–g	21.54 a–d	59	7.22 a–d	10.53 a	75
HN	9.65 g	19.61 a–e	72*	5.03 cd	9.81 ab	344*
LLR395	17.05 b–g	20.84 a–d	106	5.33 cd	6.91 a–d	49
KDML105	14.33 d–g	27.35 a	85*	6.58 a–d	8.14 a–d	58
KG	9.53 g	18.12 b–f	143*	7.80 a–d	6.28 b–d	17
LPT	12.23 e–g	10.46 fg	122	4.28 d	9.78 ab	198*
PK	18.77 b–f	21.86 a–d	46	5.43 cd	8.36 a–d	108
RD73	12.43 e–g	19.52 a–e	68	5.93 b–d	9.05 a–c	151
TSKC1–144	24.52 a–c	25.15 ab	64	6.84 a–d	9.79 ab	46
Mean	15.86	20.33	87	6.14	8.73	107

Table S9. Growth parameters including plant height and dry weight (DW) of total leaf (total leaf DW), green leaf (GL DW) and senescent leaf (SL DW) of ten rice genotypes at final harvest (110 DAG). Plants were grown under control condition and salt stress. Different lowercase letters indicate significant differences ($p < 0.05$ and $p < 0.01$) among rice genotypes and between salinity treatments. Mean % changes from control values are displayed, with significant differences between control and salt stress treatments are denoted by *. Data are Mean ± SE (n = 4).

Rice genotype	Plant height (cm)			Total leaf DW (g plant^{-1})			GL DW (g plant^{-1})			SL DW (g plant^{-1})		
	Control	Stress	%	Control	Stress	%	Control	Stress	%	Control	Stress	%
GMS	151.5 b	128.7 f–i	−15*	4.21 d–i	5.68 bc	40*	2.69 d–f	2.53 d–f	−1	1.52 f–h	3.14 ab	116*
HDN	143.5 b–d	121.7 h–k	−15*	4.05 e–i	5.30 b–f	36	2.93 c–e	2.91 de	4	1.12 gh	2.39 cd	144*
HN	138.7 c–f	120.2 i–k	−13*	3.19 i	3.27 hi	5	2.19 e–g	1.42 g	−30	1.00 gh	1.85 d–f	86*
LLR395	104.2 l	83.2 m	−20*	3.85 g–i	4.82 c–g	28	2.37 e–g	1.38 g	−39	1.48 f–h	3.44 a	143*
KDML105	131.5 e–h	119.5 i–k	−9*	3.58 g–i	4.63 c–h	31	2.34 e–g	1.85 e–g	−19	1.24 f–h	2.78 a–c	130*
KG	152.7 b	115.0 kl	−24*	7.27 a	6.60 ab	−5	6.22 a	4.07 bc	−31*	1.05 gh	2.53 b–d	169*
LPT	151.7 b	115.7 jk	−24*	5.51 b–d	5.36 b–e	−2	4.64 b	2.84 de	−38*	0.87 h	2.51 b–d	192*
PK	180.2 a	149.5 bc	−17*	4.96 c–g	6.50 ab	38*	3.29 cd	4.08 bc	31	1.67 e–g	2.41 cd	54*
RD73	134.7 d–g	127.0 g–j	−6	3.90 f–i	4.14 d–i	11	2.53 d–g	1.77 f–g	−27	1.36 f–h	2.37 c–e	88*
TSKC1–144	142.2 b–e	138.7 c–f	−2	3.64 g–i	4.06 e–i	14	2.64 d–f	1.67 fg	−36	1.00 gh	2.39 cd	165*
Mean	143.12**	121.95 B	−15	4.42	5.04	20	3.19	2.46	−19	1.23 B	2.58*	129

Table S9 (cont.). Stem DW, root DW, gain, and total DW of ten rice genotypes at final harvest (110 DAG). Plants were grown under control condition and salt stress. Different lowercase letters indicate significant differences ($p < 0.05$ and $p < 0.01$) among rice genotypes and between salinity treatments. Mean % changes from control values are displayed, with significant differences between control and salt stress treatments are denoted by *. Data are Mean ± SE (n = 4).

Rice	Stem DW (g plant^{-1})			Root DW (g plant^{-1})			Grain (g plant^{-1})			Total DW (g plant^{-1})		
**genotype**	**Control**	**Stress**	**%**	**Control**	**Stress**	**%**	**Control**	**Stress**	**%**	**Control**	**Stress**	**%**
GMS	13.41 c–e	9.55 e–g	−28	2.64 c	2.21 c	−12	9.77 b–d	4.15 ef	−58*	30.04 c	21.60 d–f	−28*
HDN	13.93 cd	9.81 d–f	−19	1.83 c	1.88 c	10	9.44 b–d	2.36 fg	−75*	29.27 c	19.36 e–g	−31*
HN	9.89 d–f	5.52 gh	−36*	1.96 c	1.51 c	0	9.23 b–d	2.18 fg	−76*	24.29 c–e	12.50 h	−48*
LLR395	6.96 f–h	4.95 h	−28	1.86 c	1.78 c	−4	13.68 a	1.85 fg	−86*	26.35 cd	13.42 gh	−48*
KDML105	9.24 fg	7.29 f–h	−20	1.71 c	1.44 c	−14	12.23 ab	1.38 fg	−89*	26.77 cd	14.76 gh	−44*
KG	34.64 a	10.24 d–f	−69*	5.84 a	2.34 c	−47*	7.01 de	0.46 g	−93*	54.77 a	19.65 e–g	−64*
LPT	28.16 b	9.29 e–g	−66*	4.04 b	2.29 c	−44*	7.82 cd	0.73 fg	−91*	45.54 b	17.68 e–h	−61*
PK	10.92 d–f	15.92 c	50*	2.41 c	2.32 c	−2	10.86 a–c	2.25 fg	−60*	29.17 c	27.00 cd	−3
RD73	10.62 d–f	6.85 f–h	−34	1.98 c	1.42 c	−29	11.04 a–c	2.71 fg	−60*	27.55 cd	15.13 f–h	−41*
TSKC1–144	15.09 c	7.02 f–h	−54*	2.19 c	1.69 c	−21	7.50 c–e	3.27 fg	−53*	28.44 c	16.05 f–h	−44*
Mean	15.29**	8.64 B	−30	2.64*	1.89 B	−16	9.86**	2.13 B	−74	32.22**	17.72 B	−41

Table S10. One-way ANOVA showing levels of significant differences ($p < 0.05$ and $p < 0.01$) for score values, SPAD index, plant height and total biomass of 24 rice genotypes grown under the non-saline and saline conditions at the seedling stage.

Parameter	Water condition (W)					Rice genotype (G)					W x G				
	df	SS	MS	*F*-test	*P*-value	*df*	SS	MS	*F*-test	*P*-value	*df*	SS	MS	*F*-test	*P*-value
Score at 7 DAS	1	721.0	721.06	190.4	0.0002	23	62.73	2.728	2.34	0.001	23	62	2.72	2.34	0.0010
Score at 10 DAS	1	1278	1278.8	2704.1	0.0000	23	145.7	6.34	4.68	0.000	23	145	6.34	4.68	0.0000
SPAD	1	4075	4075.5	41.20	0.0030	23	2771	120.5	2.99	0.000	23	2465	107.2	2.66	0.0002
Plant height	1	39501	39501	164.5	0.0002	23	11013	478.9	25.39	0.000	23	1154	50.2	2.66	0.0002
Total biomass	1	3.050	3.0504	50.80	0.0020	23	0.599	0.026	10.62	0.000	23	0.211	0.009	3.74	0.0000

Table S11. One-way ANOVA showing levels of significant differences ($p < 0.05$ and $p < 0.01$) for SPAD index, total chlorophyll (chl), chl a, chl b, chl a/b, effective quantum yield of PSII photochemistry (Φ PSII), electron transport rate (ETR), net photosynthesis rate (Pn, stomatal conductance (gs), transpiration rate (Tr), intrinsic water use efficiency (WUEi), water use efficiency (WUE), and the intercellular to ambient CO_2 ratio (Ci/Ca) at light intensity 1,200 μmol (photon) m^{-1} s^{-1}, relative water content (RWC), osmotic potential (OP), osmotic adjustment (OA), electrolyte leakage (EL), malondialdehyde (MDA), total sugar, starch content, plant height and dry weight (WD) of total leaves, green leaves (GL), senescent leaf (SL), stem, root, grain, and total DW at the reproductive growth stage.

Parameter	Water condition (W)					Rice genotype (G)					W x G				
	df	SS	MS	*F*-test	*P*-value	*df*	SS	MS	*F*-test	*P*-value	*df*	SS	MS	*F*-test	*P*-value
SPAD	1	8E-29	8E-29	000	1.000	9	199.2	22.13	8.36	0.000	9	53.28	5.920	2.24	0.033
Total Chll	1	0.370	0.370	1.50	0.308	9	1.037	0.115	1.02	0.438	9	1.390	0.154	1.36	0.227
Chl *a*	1	0.012	0.012	1.44	0.316	9	0.023	0.002	0.74	0.672	9	0.043	0.004	1.4	0.209
Chl *b*	1	0.247	0.247	0.78	0.442	9	1.059	0.117	0.96	0.481	9	1.675	0.186	1.52	0.164
Chl *a*/*b*	1	0.042	0.042	0.18	0.701	9	0.451	0.050	0.67	0.731	9	1.052	0.116	1.56	0.151
Φ PSII	1	0.021	0.021	138.2	0.001	9	0.019	0.002	2.81	0.009	9	0.036	0.004	5.21	0.000
ETR	1	7891	7891	40.57	0.008	9	4939	548.7	2.96	0.006	9	8659	962.2	5.19	0.000
Pn	1	1371	1371	870.6	0.000	9	116.3	12.92	2.47	0.019	9	104.3	11.59	2.22	0.035
gs	1	0.289	0.289	390.4	0.000	9	0.062	0.006	2.26	0.032	9	0.034	0.003	1.26	0.278
Tr	1	194.8	194.8	615.5	0.000	9	11.95	1.329	1.84	0.083	9	6.25	0.694	0.96	0.483
WUEi	1	9064	9063	6.92	0.078	9	49542	5504	4.73	0.000	9	20657	2295	1.97	0.061
WUE	1	2.888	2.888	3.22	0.171	9	45.41	5.045	7.54	0.000	9	26.57	2.9523	4.41	0.000
Ci/Ca	1	0.154	0.154	7.5	0.072	9	0.828	0.092	4.79	0.000	9	0.374	0.041	2.16	0.040

Parameter	Water condition (W)					Rice genotype (G)					W x G				
	df	SS	MS	*F*-test	*P*-value	*df*	SS	MS	*F*-test	*P*-value	*df*	SS	MS	*F*-test	*P*-value
RWC	1	251.2	251.2	8.77	0.059	9	192.7	21.41	1.88	0.075	9	137.5	15.28	1.34	0.238
OP	1	25.95	25.95	343.4	0.000	9	3.166	0.351	3.42	0.002	9	2.946	0.327	3.18	0.004
OA	1	17.15	17.15	314.0	0.000	9	2.497	0.277	3.12	0.004	9	2.182	0.242	2.73	0.011
EL	1	115.2	115.2	18.27	0.024	9	235.9	26.21	1.8	0.091	9	126.8	14.08	0.96	0.479
MDA	1	349.8	349.7	1.3	0.337	9	3028	336.4	4.27	0.000	9	587.4	65.27	0.83	0.593
Na^+ content	1	95.24	95.24	753.4	0.000	9	4.83	0.537	14.7	0.000	9	5.04	0.560	15.35	0.000
K^+ content	1	0.187	0.187	219.9	0.001	9	6.96	0.773	9.18	0.000	9	3.396	0.377	4.48	0.000
Na^+/K^+ ratio	1	36.95	36.95	478.0	0.0002	9	3.65	0.4062	18.1	0.000	9	3.59	0.399	17.8	0.000
Total sugar	1	398.4	398.4	5.15	0.108	9	1203	133.6	4.07	0.001	9	554.9	61.66	1.88	0.075
Starch	1	134.1	134.1	6.76	0.080	9	42.59	4.733	0.61	0.782	9	67.97	7.552	0.97	0.471
Plant height	1	8967	8967	510.1	0.000	9	2188	2431	38.3	0.000	9	2346	260.6	4.11	0.001
Total leaf DW	1	7.685	7.684	1.53	0.305	9	83.86	9.318	12.1	0.000	9	9.988	1.109	1.45	0.190
GL DW	1	10.65	10.65	6.54	0.083	9	83.24	9.249	23.0	0.000	9	12.98	1.443	3.59	0.002
SL DW	1	36.44	36.44	30.2	0.012	9	7.009	0.778	3.88	0.001	9	2.678	0.297	1.48	0.179
Stem DW	1	882.0	882.0	39.04	0.008	9	1896	210.7	26.9	0.000	9	1346	149.6	19.1	0.000
Root DW	1	11.51	11.51	12.81	0.037	9	45.77	5.086	7.39	0.000	9	21.27	2.363	3.43	0.002
Grain weight	1	1193	1193	380.2	0.000	9	114.6	12.74	1.91	0.070	9	93.94	10.44	1.56	0.150
Total DW	1	4207	4207	84.9	0.003	9	2498	277.6	13.5	0.000	9	1676	186.2	9.1	0.000

Table S12. Physicochemical properties of soil and water in this experiment.

Trait	Soil physicochemical properties	
Physical properties at pre-planting		
Sand (%)	58.48	
Silt (%)	30.10	
Clay (%)	11.42	
Soil Texture	Sandy Loam	
Chemical properties at pre-planting		
Total N (%)	0.03	
Available P (mg kg^{-1})	22.50	
Exchangeable K (mg kg^{-1})	95.76	
Organic matter (%)	0.85	
CEC (cmol kg^{-1})	6.85	
pH (1:1 H_2O)	6.35	
EC soil (dS m^{-1})	0.08	
**Crop growth stage**	**EC water (dS m^{-1})**	
	**Control**	**Stress**
Seedling stage	0.080 ± 0.01	15.1 ± 0.30
Early booting to flowering stage	0.082 ± 0.04	15.2 ± 0.50
Milky stage to final harvest	0.074 ± 0.05	12.3 ± 0.44

Table S13. The salt injury scores of rice genotypes at the seedling stage.

Score	Observation	Tolerance level
1	Normal growth	Highly tolerance (HT)
3	Nearly normal growth;<br>leaf tips or leaves whitish and rolled	Tolerance (T)
5	Growth severely retarded;<br>Most leaves rolled; only a few are elongating	Moderately tolerance (MT)
7	Complete cessation of growth;<br>Most leaves dry; some plants drying	Sensitive (S)
9	Almost all plant dead or dying	Highly sensitive (HS)
